# Establishment of Labor Epidural Analgesia Service and its Assessment: An Experience in a Hospital of a Middle-Income Country

**DOI:** 10.7759/cureus.55322

**Published:** 2024-03-01

**Authors:** Anita Djurdjevic Svraka, Dragan Svraka, Dejan Pejic, Vladimir Mrdja

**Affiliations:** 1 Faculty of Medicine, University of Banja Luka, Banja Luka, BIH; 2 Anesthesiology, Resuscitation, and Intensive Care, General Hospital Gradiska, Gradiska, BIH; 3 Anesthesiology and Critical Care, University Clinical Center of Republika Srpska, Banja Luka, BIH; 4 Obstetrics and Gynaecology, General Hospital Gradiska, Gradiska, BIH

**Keywords:** patient safety, health public, labor pain management, establishment of procedure, regional anesthesia

## Abstract

Objectives: Even though the idea of painless birth is more than 100 years old, it is still underrepresented in some parts of the world despite progress in science, education, anesthesia, spinal and epidural needles, development of catheters, new drugs, and infusion pumps. Maternal care should basically be the safety of the patient (in this case two patients) and also provide all kinds of protection in the form of a multidisciplinary team with an anesthesiologist, especially when it comes to pain therapy and anesthesia for women in labor. In this direction, our hospital departments with low or moderate volume of annual births made the decision to educate ourselves for painless childbirth and contribute more to the care of women in labor. The enthusiasm and dedication of our clinical team prevailed and today we have a high standard of labor epidural service. The aim of this study was to evaluate the establishment of the epidural labor service by comparing the effects of epidural analgesia on labor pain and the course of labor. The secondary objectives were to compare satisfaction with epidural analgesia and the impact of epidural analgesia on the delivery mode.

Material and methods: This was a prospective observational hospital-based study conducted on 100 patients after the establishment of epidural service. Parturients who were signed to receive epidural analgesia formed Group A and parturients who did not request epidural analgesia formed Group B. All parturients are induced in the delivery room with a Bishop score of 5 or higher.

Results: Pain intensity measured through the visual analog scale of pain (VAS score) was significantly lower in Group A (n* = *46) compared to Group B (n* = *50) at measured points of time (p<0.001)*. *There was a fall in the mean VAS score in Group A from 7.94 to 3.86 within 20 minutes of the bolus dose and starting a continuous infusion. Labor progress according to the Bishop score and till the end of the second stage of labor, or to the transfer to the operating theatre, according to the monitored time in Group A was 176 minutes and it was lower in Group B with 155 minutes; however, by test of linearity we do not gain significance (p = 0.2*)*. There were eight parturients in each group (17% vs 16%) who were indicated for surgical delivery. According to Pearson's correlation test for the outcome of labor between parturients receiving epidural analgesia and parturients without epidural analgesia (p = 0.8), we cannot say that epidural analgesia in labor is correlated with the outcome of surgical delivery.

Conclusion: Establishing a new hospital procedure such as an epidural painless service for childbirth in low or moderate-volume settings of annual births is very challenging. By evaluating epidural labor services in our hospital, we created the best environment for continuous improvement and long-term efficacy and safety of our analgesic techniques aimed at providing excellent care to mothers and their babies.

## Introduction

The most difficult step in establishing the standard of providing pain therapy and anesthesia for women in labor is the education of the team and going against the traditional way of thinking that minimizes the importance of pain during childbirth. Pain is not only an unpleasant feeling, pain is also fear, uncertainty, and helplessness, and therefore women in labor should receive pain therapy. 

We had a non-adequately equipped unit and dedicated leadership whose role was to ensure high standards while developing an epidural service in a country with limited resources. Besides University Clinical Centers in Bosnia and Herzegovina, in the last 10 years, some hospitals that have between 800 and 2000 births per year achieved their epidural service goals, while others did not. However, no official statistics have been published. 

In low- and middle-income countries (LMICs), vulnerable patient populations, such as pregnant women, still do not have access to high-standard healthcare. Consequently, there is a large discrepancy when it comes to analgesia for labor. Thus, while in the United States, neuraxial labor analgesia use ranges from 66% to 82% [1], one study estimated that only 2.2% of parturients at a South African public hospital received neuraxial labor analgesia [2]. Even though the idea of a painless birth is more than 100 years old [3], it is still underrepresented in some parts of the world despite tremendous progress in science, education, anesthesia, spinal and epidural needles, development of catheters, new drugs, and infusion pumps.

Maternal care not only includes the safety of the patient (in this case two patients, mother and baby) but also protection in the form of a multidisciplinary team with an anesthesiologist, especially when it comes to pain therapy and anesthesia for obstetric patients. In this study, our aim was to monitor the success of establishing the epidural analgesia procedure during labor and to show the impact of epidural analgesia on the possible prolongation of the delivery time and the impact on the mode of delivery.

## Materials and methods

This was a hospital-based prospective observational study conducted on 100 nulliparous women at term with a single fetus in a cephalic presentation in active labor. The study started with the beginning of epidural service in General Hospital Gradiska, Gradiska, Bosnia and Herzegovina, in September 2016 and was conducted till June 2019. All parturients signed a consent. The study was approved by the Ethics Committee of the General Hospital Gradiska (approval number: 09/022/2016, dated September 7, 2016).

Nulliparous patients who were willing to receive epidural analgesia formed Group A (n=50) and nulliparous patients who refused epidural analgesia formed Group B (n=50). Parturients who were at the beginning of active labor and after obstetric indication were evaluated as candidates for vaginal birth were included. The age criterion for inclusion was ≥18 years but there was no body mass index limit. All women in labor were at term, ≥38 weeks of pregnancy. Patients with contraindications to the placement of epidural catheters or local anesthetics injections are excluded.

The protocol dictated that the patient be admitted to the delivery room only when a Bishop score of 5 or higher was reached, and the epidural catheter was placed when the cervix was dilated 4-5 cm, after entering the delivery room. The Bishop score includes cervical dilatation, effacement, station of the fetal head, consistency of the cervix, and position of the cervix, which is monitored by the obstetrician. The epidural analgesia protocol after a successfully placed epidural catheter started with a bolus of 10 ml of a 50 ml mixture of 0.125% bupivacaine and 4 µg/ml of fentanyl. After the bolus injection, the infusion pump without the patient control button was started at a rate of 5-10 ml/hour depending on the pain visual analog scale (VAS) (scale from 1 to 10). The goal was to have pain sensitivity at 2-4 on the scale. With a pain assessment of 5 or more, an added bolus of 5-10 ml would be needed if the pump was at 10 ml, or if the pump was set at 5 ml/hour, the boost would be increased to 10 ml/hour, with or without bolus prescribed by the anesthesia staff. During the active labor, parturients were non-invasively hemodynamic and respiratory monitored.

All patients were prescribed oxytocin infusion at one point. Most often, the infusion of 5 IU of oxytocin in 500 ml of 5% dextrose solution started upon entering the delivery room, but it depended on the moment of amniotomy and dinoprostone application in some cases. The infusions went in individual doses of 2-30 ml/minute until the desired strength of contraction was reached, according to the findings and assessment of the obstetrician. Oxytocin infusion was interrupted on several occasions, e.g. at the time of epidural catheter placement, sometimes vaginal examination, characteristic of cardiotocographic findings. Basically, it can be said that an individual minimum effective dose of oxytocin was always used in both groups of parturients. 

The survey on patient satisfaction with epidural analgesia for labor was only related to the group that chose epidural as an option (Group A). The questionnaire was filled out immediately after delivery while the patients were still in the delivery room for observation after delivery. The survey was conducted based on three simple survey questions: very satisfied, satisfied, and dissatisfied with epidural analgesia.

Statistical analysis was performed using IBM SPSS Statistics for Windows, Version 25.0 (Released 2017; IBM Corp., Armonk, New York, United States). For continuous data, mean ± standard deviation or median (interquartile range) was used as applicable. The categorical data was compared using Chi-Square test. To test correlation between variables, we used Pearson's Correlation test.

## Results

Placement of epidural catheter failed in four (8%) of the 50 patients in Group A. Parturients did not differ significantly by mean age (Group A 29.2±6.4 years vs Group B 28.5±5.4 years; Mean ±SD) (p = 0.5). Similar findings were seen with weeks of gestation (39.3±1.9 vs 39.2±0.6; Mean ±SD) (p = 0.1) and BMI (29.6±4.3 vs 28.3± 4.0 24±3.0; Mean ±SD) (p = 0.7). The total VAS score from the start of measuring the time of delivery in the delivery room (starting from consent or refusal of epidural painless delivery until the end of the second stage of labor), which was recorded in the table every half hour, was significantly lower in Group A (24±3.0; Mean ±SD) compared to Group B (44±9.0; Mean ±SD), (p<0.001) (Table 1).

**Table 1:** Descriptive and general monitored variables for Group A (with epidural analgesia for labor) and Group B (without epidural analgesia for labor). Data are presented as  n (number of patient), Mean±SD (standard deviaton) *Two-sample independent t-test (Significance level p=0.05); ** Chi-Square test (Significance level of p=0.05)

Variables	Group A (n = 46) (Mean±SD)	Group B (n = 50) (Mean±SD)	P
Age (years)	29.2± 4.6	28.5 ±5.4	0.5*
Gestation (weeks)	39.3±1.9	39.2±0.6	0.1*
BMI (kg/m^2^)	29.6±29.6	28.3±4.0	0.7*
VAS	23±3.0	44±9.0	<0.001**
Duration (minutes)	176±8.0	155±8.0	0.2*

There was a fall in mean VAS score in group A from 7.94±4.6 to 3.86±4.0 (Mean ±SD) within 20 minutes with the bolus dose and starting a continuous infusion. Labor epidural analgesia for active labor parturients in the delivery room resulted in better pain management during labor and had a large impact on patient satisfaction during labor. Only one parturient in Group A was not satisfied with epidural analgesia despite the VAS score measured being lower than 5 after epidural analgesia started.

**Figure 1:**
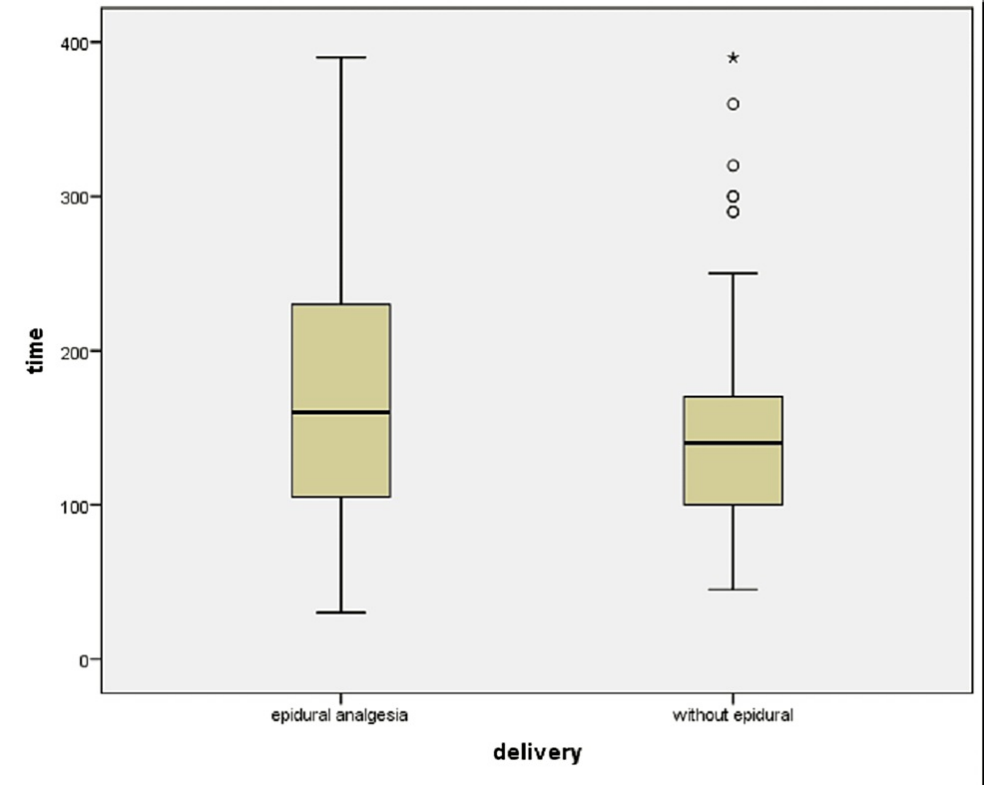
Box-plot of labor duration by time in minutes between groups (with or without epidural analgesia). Measured from entering the delivery room (Bishop score ≥5 ) to the end of the second stage of labor for vaginal delivery (Bishop score 13 with fetal expulsion), or till going to the operation theater for surgical delivery.

Labor duration according to the progression of Bishop score till the end of the second stage of labor or to the transfer to the operating theatre, according to the monitored time in minutes was 176 minutes (176±8.0; Mean±SD) in Group A; it was lower in Group B (155±8.0; Mean±SD) but not significant by t-test (p = 0.2) (Table 1, Figure 1).

**Figure 2:**
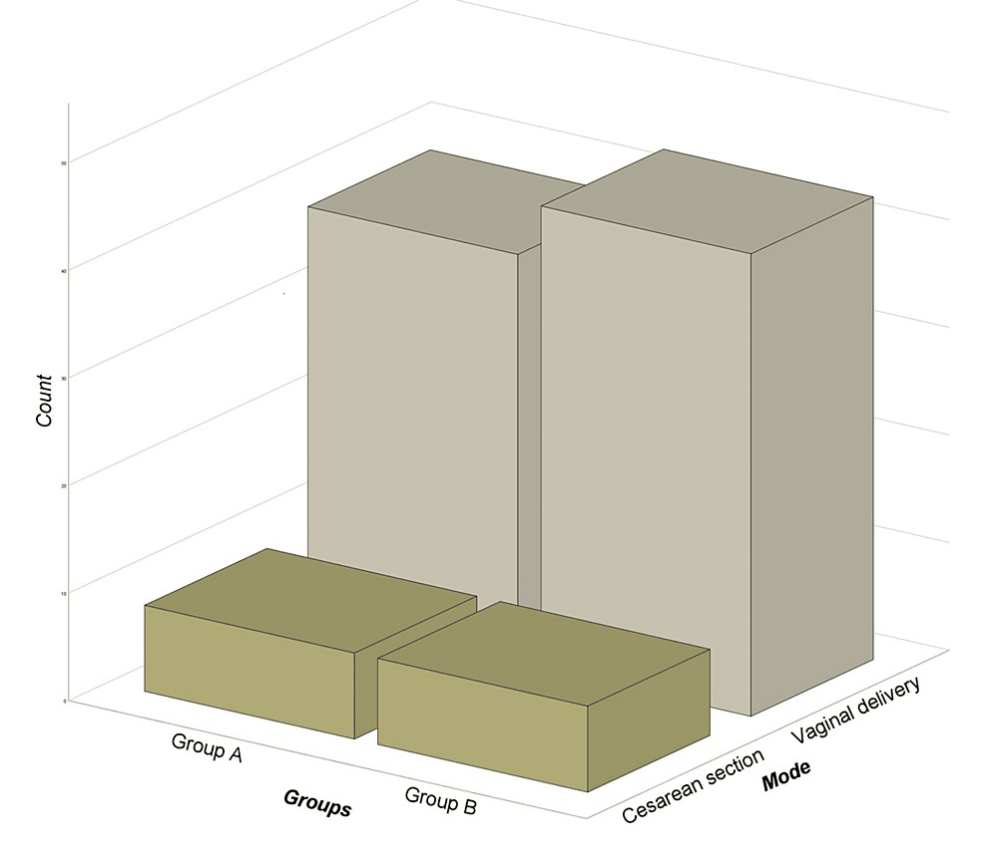
Vaginal and surgical deliveries in the two groups of patients, Group A with epidural analgesia and Group B without epidural analgesia.

Eight parturients in each group were indicated for surgical delivery. According to Pearson's correlation test for the outcome of labor between parturients receiving epidural analgesia and parturients without epidural analgesia (p = 0.8), we cannot say that epidural analgesia in labor is correlated with the outcome of surgical delivery (Figure 2).

## Discussion

Introducing the procedure of painless delivery with epidural analgesia when there are no nationalized standard procedures can be very challenging. There are no published studies dealing with the subject in the region of Bosnia and Herzegovina. From a professional point of view, the focus would be on gaining the experience of placing an epidural catheter in obstetric patients, especially when the procedure is introduced for the first time in the hospital. Labor epidural analgesia failure rate is 9-12% and can be multifactorial [4]. Poor insertion technique (multiple needle passes), needle mal-placement (off midline), inadequate loading dose and/or infusion rate, catheter migration, and rapid labor progression are the most common causes of labor epidural failure. Whatever the reason for analgesia failure, we must always be guided by the fact that the patient's safety is most important. Immediate identification of intrathecal and intravascular catheters is vital. In our case, epidural failure was 8% and the reason was the impossibility of identifying the epidural space, which is common for beginners. The epidural technique is complex, but obstetric patients also have their own specificities, such as obesity, a smaller diameter of epidural space due to increased intra-abdominal pressure, and an inability to palpate the bony landmarks [5,6].

Epidural analgesia for childbirth is a highly effective pain therapy as reported in all published studies, while the satisfaction with this method of treatment depends on the method of the survey in terms of scoring satisfaction from 1 to 10, or simply according to the declaration at the end of the birth: very satisfied, satisfied, not satisfied [7-9]. In our sample, there was one unsatisfied patient, despite the successful epidural analgesia as measured by the VAS score, compared to all other patients who were highly satisfied, which suggests that the patient's answer regarding her satisfaction is highly subjective.

One of the perennial dilemmas is whether epidural analgesia for labor leads to prolonged vaginal delivery. The results of the studies differ, whether because of the women in labor being nulliparous or due to ignoring the impact of labor induction [10-12]. Induction of labor by oxytocin and prostaglandins is dose- and route-dependent and should be one of the monitoring parameters precisely because of this but in practice is complex. The RIPE (Results of Induction of Labor with Prostaglandins E1 and E2) study of Socha et al. conclude that patients who received the dinoprostone vaginal insert required statistically significantly more oxytocin administration than patients who received the misoprostol vaginal insert [13]. A study by European and South African authors that looked at oxytocin administration regimens in 12 countries concluded that variations in oxytocin regimens for induction and augmentation of labor are inexplicable [14]. The length of labor is also affected by many other factors such as mental and physical unpreparedness for childbirth, unrecognized cephalopelvic disproportion (CPD), change in the fetal position, and poor expulsive efforts or exhaustion of the mother. Weak expulsive efforts resulting from conductive analgesia or sedation speak in favor of inadequate monitoring and management of painless delivery.

The effect of epidural analgesia on labor prolongation and labor outcome is also the subject of studies and meta-analyses. There are studies with significant results that epidural analgesia during labor has no effect on the outcome of labor, but prolongs labor, especially the second stage of labor [11,12]. A cohort study of 23,183 deliveries (both spontaneous and induced) by Penuela et al. concluded that epidural analgesia was an independent risk factor for instrumental and surgical delivery and abnormal fetal head position at delivery [15]. Our observations on the impact of epidurals on delivery outcomes, compared to the sample size of the aforementioned studies on the same topic, are still based on a small number of patients, as far as surgical delivery is concerned, while instrumental delivery was not recorded in our sample and is very rare in general in our practice.

As with most studies in obstetric patients, the design of the current study is subject to limitations. First, the study was not randomized. Second, the sample size was small, which in the case of sampling and randomization could lead to selection bias. Third, the study is single-centered. The lack of consistency in oxytocin therapy and individually administered doses of oxytocin could have influenced the results of the study.

## Conclusions

Establishing a new procedure such as epidural labor analgesia in hospital settings with a low or moderate volume of annual births is very challenging. From our study, it could be concluded that we carried out the procedure successfully at the beginning of establishing an epidural analgesia labor. Although by monitoring the time of delivery in the delivery room, we prolonged the delivery of patients with an epidural catheter, we cannot conclude that this had an effect on the increased number of cesarean sections. By evaluating epidural labor services in our hospital, we created the best environment for continuous improvements and long-term efficacy and safety of our analgesic techniques aimed at providing excellent care to parturients.
